# Cost-effectiveness of GLP-1 receptor agonists versus insulin for the treatment of type 2 diabetes: a real-world study and systematic review

**DOI:** 10.1186/s12933-020-01211-4

**Published:** 2021-01-19

**Authors:** Chen-Yi Yang, Ying-Ren Chen, Huang-Tz Ou, Shihchen Kuo

**Affiliations:** 1 Institute of Clinical Pharmacy and Pharmaceutical Sciences, College of Medicine, National Cheng Kung University, 1 University Road, Tainan, Taiwan; 2 Department of Pharmacy, College of Medicine, National Cheng Kung University, Tainan, Taiwan; 3 Department of Pharmacy, National Cheng Kung University Hospital, Tainan, Taiwan; 4 Division of Metabolism, Endocrinology & Diabetes, Department of Internal Medicine, University of Michigan, Ann Arbor, MI USA; 5 Michigan Center for Diabetes Translational Research, University of Michigan, Ann Arbor, MI USA

**Keywords:** Cost-effectiveness analysis, GLP-1 receptor agonist, Insulin, Type 2 diabetes, Systematic review

## Abstract

**Background:**

To conduct a real-word-study-based cost-effectiveness analysis of a GLP-1 receptor agonist (GLP-1RA) versus insulin among type 2 diabetes patients requiring intensified injection therapy and a systematic review of cost-effectiveness studies of GLP-1RAs versus insulin.

**Methods:**

Individual-level analyses incorporating real-world effectiveness and cost data were conducted for a cohort of 1022 propensity-score-matched pairs of GLP-1RA and insulin users from Taiwan’s National Health Insurance Research Database, 2007–2016. Study outcomes included the number needed to treat (NNT) to prevent one case of clinical events, healthcare costs, and cost per case of event prevented. Costs were in 2019 US dollars. Analyses were performed from a third-party payer and healthcare sector perspectives. Structured systematic review procedures were conducted to synthesize updated evidence on the cost-effectiveness of GLP-1RAs versus insulin.

**Results:**

Over a mean follow-up of 2.3 years, the NNT using a GLP-1RA versus insulin to prevent one case of all-cause mortality and hospitalized hypoglycemia was 57 and 30, respectively. Using GLP-1RAs instead of insulin cost US$54,851 and US$29,115 per case of all-cause mortality and hospitalized hypoglycemia prevented, respectively, from the payer perspective, and saved US$19,391 and US$10,293, respectively, from the healthcare sector perspective. Sensitivity analyses showed that the probability of using GLP-1RAs versus insulin being cost-effective for preventing one case of all-cause mortality or hospitalized hypoglycemia ranged from 60 to 100%. The systematic review revealed a cost-effective profile of using GLP-1RAs versus insulin.

**Conclusions:**

Using GLP-1RAs versus insulin for type 2 diabetes patients requiring intensified injection therapy in clinical practice is cost-effective.

## Background

Many patients with type 2 diabetes (T2D) eventually need a greater potency of injectable glucose-lowering agents (GLAs) because T2D is a progressive disease [1, 2]. Recent guidelines by the American Diabetes Association and the European Association for the Study of Diabetes recommend that for most such patients, glucagon-like peptide-1 receptor agonists (GLP-1RAs) are preferable to insulin as the initial injection therapy and are also the preferred choice for addition to basal insulin for combination injection therapy [3]. These recommendations are based on results that showed that GLP-1RA compared with insulin therapy (basal, premixed, or intensified basal-bolus regimens) have similar or even better efficacy in HbA1c reduction and a lower hypoglycemia risk, promotes weight loss instead of weight gain, and reduces the burden of drug administration frequency and self-monitoring of blood glucose [1, 2]. Moreover, a meta-analysis of cardiovascular outcomes in trials of GLP-1RAs has revealed cardiovascular benefits in T2D patients with or without pre-existing cardiovascular diseases [4]. However, the common gastrointestinal symptoms and higher drug acquisition costs of GLP-1RAs have hindered their widespread and continuous use in real-world settings [2, 5]. In Taiwan’s National Health Insurance program, the cost of a GLP-1RA (e.g., liraglutide) can be up to 7 times that of insulin (e.g., insulin glargine) per treatment cycle (e.g., US$108.6 versus US$15.4 per month) [6]. The trade-offs among clinical effectiveness, tolerability, and cost for GLP-1RA use have highlighted the importance of a cost-effectiveness analysis (CEA) of GLP-1RA versus insulin therapy in real-world clinical practice [2].

CEA results can support treatment prioritization, facilitate healthcare reimbursement policy formulation, and optimize healthcare resource allocation. The incorporation of real-world effectiveness and cost data into CEA complements the evidence derived from clinical trials and ensures that the results will be relevant for the real-life healthcare decision-making context [7, 8]. However, most CEAs of GLP-1RA versus insulin therapy for T2D patients have been model-based (e.g., Markov modeling simulation) analyses that used data mainly derived from clinical trials that assessed short-term drug efficacy in terms of biomarker changes among highly selective and homogenous patient populations [9–28]. Such approaches greatly affect the generalizability of study results to real-world settings and raise concerns about the validity of projecting the results to long-term outcomes.

There has been no real-world-study-based CEA of GLP-1RAs versus insulin use among T2D patients who require intensified injection therapy. The present study conducts a real-world-study-based CEA of GLP-1RAs versus insulin using rigorous methodologies to ensure the representativeness of the study cohort in relation to real-world patients and the comparability between study groups for valid effectiveness and cost estimates used in the CEA. In addition, a systematic review of CEAs of GLP-1RAs versus insulin among T2D patients was performed to provide up-to-date evidence about the use of GLP-1RAs versus insulin in terms of cost effectiveness.

## Research design and methods

This study was approved by the Institutional Review Board of National Cheng Kung University (A-EX-106-013). The CEA was performed in compliance with recommendations by the Second Panel on Cost-Effectiveness in Health and Medicine [29]. An impact inventory (Additional file 1: Table S1) was used to summarize the effectiveness and cost consequences associated with GLP-1RAs and insulin therapies from a third-party payer (payer hereafter) and healthcare sector perspectives. The five-step study framework of this CEA is illustrated in Additional file 1: Fig. S1. The steps, described in detail below, are (1) cohort identification, (2) effectiveness estimation for clinical outcomes of interest, (3) cost estimation, (4) base-case analysis, and (5) sensitivity analyses. The CEA results in the base-case and sensitivity analyses are summarized as incremental cost-effectiveness ratios (ICERs). The Consolidated Health Economic Evaluation Reporting Standards checklist was used as a guide for data reporting (Additional file 1: Table S2).

### Data source and cohort identification

Study subjects were identified from Taiwan’s National Health Insurance Research Database (NHIRD) in the period 2007–2016. The NHIRD includes population-based claims data of the National Health Insurance (NHI) program, a mandatory-enrollment, single-payment system that covers over 99% of Taiwan’s population. This database includes patient demographics and all medical service records of disease diagnoses, procedures or surgeries, and prescriptions and medical supplies reimbursed by the NHI program [30].

Patients with newly diagnosed T2D during 2008–2015 were identified from the NHIRD. Stable users of a GLP-1RA or insulin in 2011–2015 were further selected; this period was chosen because the reimbursement for GLP-1RAs in Taiwan’s NHI program began in 2011. Stable drug users were defined to exclude potential confounding due to the short-term use of study drugs. The index date was defined as the date of the beginning of the stable use of a GLP-1RA or insulin. Patients were followed up from the index date until death, loss to follow-up, development of a clinical outcome of interest, or the end of 2016, whichever came first, as the intention-to-treated (ITT) analysis scenario. Details of the study cohort selection are provided in Additional file 1: Fig. S2. The comparability of patient characteristics between two study groups was achieved by the three-step matching on (1) the index date, (2) prior exposure to GLAs in the year before the index date, and (3) patient demographic and clinical characteristics (e.g., diabetes severity, comorbidities, co-medications for cardiovascular diseases [CVDs]) using the propensity score matching approach [6]. After the matching, there were 1022 matched pairs of GLP-1RA and insulin users with no significant between-group difference in the baseline characteristics except for age, hyperlipidemia, and neuropathy (Additional file 1: Table S3).

### Effectiveness estimation for clinical outcomes of interest

The clinical outcomes included the composite CVD, three-point major adverse cardiovascular events (MACE), fatal CVD, all-cause mortality, and hospitalized hypoglycemia. Their operational definitions are detailed in Additional file 1: Table S4.

Number needed to treat (NNT) measures were applied to estimate the treatment effectiveness for clinical outcomes in this CEA. They can be interpreted as the average number of T2D patients who would need to be treated with a GLP-1RA relative to insulin for a given follow-up period of time to prevent one case with a clinical outcome of interest; a lower absolute value of an NNT indicates a higher degree of effectiveness of a GLP-1RA versus insulin. NNT measures have great clinical relevance and have been increasingly used in health economic evaluations related to diabetes [31–35]. Understanding the relationship between CEA and NNT helps clinicians apply CEA findings in clinical practice [31].

The survival probabilities derived from the results (i.e., hazard ratios [HRs]) of multivariable Cox proportional hazard model analyses with adjustment for imbalanced patient characteristics were used to estimate the NNT measures (Eq. 1) [36]. A robust sandwich covariance matrix estimator was used in the Cox model analyses to account for the re-use of stable use sets of insulin in the matching for GLP-1RA users (Additional file 1: Fig. S2) [37]. To support the CEA, NNT measures were estimated for clinical outcomes with a statistically significant difference (*p*-value of HRs < 0.05) between study groups.

1 $${\text{NNT }} = 1/\left( {S_{GLP - 1ra} - S_{insulin} } \right) = 1/\left\{ {\left[ {S_{insulin} \left( t \right)} \right]^{hazard ratio} - S_{insulin} \left( t \right)} \right\}$$

where *S* is the survival probability based on the time *t* from the Cox model analysis.

### Cost estimation

For each patient, we used NHI claims data to measure all healthcare costs during the follow-up period between the index date and the end of 2016. These costs included diabetes-related and -unrelated medical costs paid by the payer and the copayments paid as out-of-pocket (OOP) expenses by patients. From the payer perspective, all medical costs paid by the payer related to emergency department visits, inpatient admissions, outpatient visits, and pharmacies were included, and from the healthcare sector perspective, the OOP expenses were also included in the analyses.

To account for the between-group difference in baseline healthcare costs, a regression analysis was performed to adjust for healthcare costs in the year before the index date (Eqs. 2 and 3) [35, 38]. All cost estimates were standardized to the year 2019 using the medical care component of Taiwan’s consumer price index (CPI) (https://eng.stat.gov.tw/public/data/dgbas03/bs3/english/cpiidx.xls) and then converted to 2019 US dollars using an average exchange rate of US$1:NT$30.905.

2 $${\text{Cost}}_{{{\text{follow}} - {\text{up}}_{j} }} = \alpha + \beta_{\text{baseline}} \times {\text{Cost}}_{{{\text{baseline}}_{j} }} + \beta_{\text{treatment}} \times {\text{Treatment}}_{j}$$

3 $${ \ln }\left( {{\text{Cost}}_{{{\text{adjusted}}_{j} }} } \right) = { \ln }({\text{Cost}}_{{{\text{follow}} - {\text{up}}_{j} }} ) - \beta_{\text{baseline}} \times \left( {{ \ln }({\text{Cost}}_{{{\text{baseline}}_{j} }} } \right) - { \ln }\left( {{\text{Cost}}_{\text{mean at baseline}} } \right))$$

### Base-case analysis

In the base-case CEA, the ITT scenario was applied for treatment effectiveness estimation and the follow-up period between the index date and the end of 2016 was used for cost estimation. The ICER was estimated as the incremental costs per case of the clinical event prevented when using a GLP-1RA versus insulin, and estimated by multiplying the incremental adjusted costs between GLP-1RA and insulin groups during the follow-up period by the NNT for a given significant clinical outcome from the payer and healthcare sector perspectives. The incremental adjusted costs refer to the difference in the average per-patient healthcare costs during a given follow-up period between GLP-1RA and insulin groups, with adjustment for baseline healthcare costs.

### Sensitivity analyses

Three sensitivity analyses were conducted to examine the robustness of the base-case CEA results: (1) the ITT scenario was used for treatment effectiveness estimation, and the follow-up period between the index date and the development of a clinical outcome or the end of 2016 was used for cost estimation; (2) the as-treated (AT) scenario, where patients were observed from the index date until death, loss to follow-up, development of a clinical outcome, discontinuation of the study group treatment, or the end of 2016, whichever came first, was adopted for treatment effectiveness estimation, and the follow-up period between the index date and the end of 2016 was used for cost estimation; and (3) the AT scenario was applied for treatment effectiveness estimation, and the follow-up period between the index date and the development of a clinical outcome or the end of 2016, whichever came first, was used for cost estimation.

To assess the sampling uncertainty in the ICER estimates, the nonparametric bootstrap method was applied to generate 1000 replicated estimates of incremental cost-effectiveness pairs for study patients [39], where the 95% confidence interval (CI) for ICERs was defined as the 2.5th and 97.5th ranked ICER of the 1000 replicated estimates. Cost-effectiveness acceptability curves were then obtained as a summary measure of the joint uncertainty of costs and effectiveness, which indicated the probability of cost-effectiveness at various willingness-to-pay (WTP) thresholds [40]. As suggested by the World Health Organization [41], an intervention strategy is considered cost-effective or marginally cost-effective in Taiwan if the ICER is less than one time Taiwan’s gross domestic product (GDP) per capita (US$25,893 in 2019) or three times Taiwan’s GDP per capita (US$77,679 in 2019), respectively [42] (Taiwan’s WTP norms).

### Systematic review

Details of the systematic review procedures are provided in Additional file 1: Fig. S3. They follow those in a previous review of cost-effectiveness studies related to diabetes [43, 44]. Briefly, two reviewers (Yang and Chen) independently searched for studies in PubMed and Embase from the inception of the databases to June 30, 2020, that reported the cost-effectiveness of GLP-1RAs versus insulin for T2D patients. The search strategy and key terms are listed in Additional file 1: Fig. S3. We included original research studies in English with full-text articles available. Each study was screened for eligibility by each author, with disagreements resolved by group discussion and consensus. All costs and ICERs (expressed as dollars per quality-adjusted life year [QALY] gained) were adjusted to 2019 US dollars using the overall CPI [45]. We calculated a range and median ICER for all CEAs of GLP-1RAs versus insulin and by individual GLP-1RA drugs, and then used the US [44] and Taiwan’s WTP norms to determine whether a GLP-1RA versus insulin is: (1) cost-saving (when ICER < 0), (2) cost-effective (ICER < US$50,000 [US norm] or US$25,893 [one time Taiwan’s GDP]), (3) marginally cost-effective (ICER between US$50,000 and US$100,000 [US norm] or between US$25,893 and US$77,679 [one and three times Taiwan’s GDP]), or (4) not cost-effective (ICER > US$100,000 [US norm] or US$77,679 [three times Taiwan’s GDP]).

## Results

### Effectiveness estimation

Table 1 presents the treatment effectiveness estimation based on NNT measures derived from the HR results of Cox model analyses. To facilitate the CEA, the NNT measures were only estimated for the statistically significant clinical outcomes between study groups: all-cause death (HR [95% CI]: 0.40 [0.18, 0.91]) and hospitalized hypoglycemia (0.43 [0.27, 0.69]). Compared to insulin therapy, 57 and 30 patients would need to be treated with a GLP-1RA over a mean of 2.3 years to prevent one case of all-cause mortality and hospitalized hypoglycemia, respectively.

**Table 1:** Disaggregated results of effectiveness associated with GLP-1RAs versus insulin therapy for clinical outcomes for a cost-effectiveness analysis

Outcome	Incidence rate per 1000 person-years		Mean follow-up time in years (overall)	HR and 95% CI^c^	NNT^d^
	GLP-1RAs	Insulin			
Composite CVD^a^	50.94	55.05	2.15	1.19 (0.91, 1.57)	N/A
MACE^b^	13.73	17.89	2.26	1.13 (0.68, 1.89)	N/A
Fatal CVD	2.53	3.83	2.31	1.66 (0.50, 5.51)	N/A
All-cause death	3.81	13.88	2.28	0.40 (0.18, 0.91)^*^	56.63
Hospitalized hypoglycemia	10.73	25.75	2.26	0.43 (0.27, 0.69)^**^	30.06

GLP-1RAs: glucagon-like peptide-1 receptor agonists; HR: hazard ratio; CI: confidence interval; NNT: number needed to treat; CVD: cardiovascular disease; N/A: not applicable; MACE: major adverse cardiovascular events

^a^Composite CVD included acute myocardial infarction, ischemic heart disease, heart failure, stroke, cardiogenic shock, sudden cardiac arrest, arteriosclerotic cardiovascular disease, and arrhythmia

^b^MACE denotes three-point major adverse cardiovascular events, including non-fatal myocardial infarction, non-fatal stroke, and fatal CVD

^c^The Cox model analysis was undertaken with a robust sandwich covariance matrix estimator to account for the re-use of stable use sets of insulin in the matching for GLP-1RA users

^d^The NNT estimate was only estimated for study outcomes with a statistically significant difference between two treatment groups (i.e., *p*-value of hazard ratio < 0.05). The NNT was constructed based on the calculation of survival probabilities from the Cox proportional model with the adjustment of imbalanced baseline patient characteristics shown in Additional file 1: Table S2

* *p* < 0.05, ** *p* < 0.001

### Cost estimation

Table 2 summarizes the disaggregated cost estimates over a mean follow-up period of 2.28 years. From the payer perspective, the adjusted total cost per patient for the GLP-1RA group was higher than that for the insulin group by US$969, and from the healthcare sector perspective, the adjusted total cost per patient for the GLP-1RA group was lower than that for the insulin group by US$342. The lower total cost is mainly attributable to the reduced costs for emergency visits and inpatient admissions in the GLP-1RA group.

**Table 2:** Disaggregated results of costs per subject over a mean follow-up period of 2.28 years for a cost-effectiveness analysis

	GLP-1RAs (n = 1022)	Insulin (n = 1022)	ΔCost
Third-party payer perspective			
Baseline costs	2119	2672	− 552
Third-party payer costs	6969	7813	− 844
Third-party payer costs (adjusted^a^)	9189	8221	969
Healthcare sector perspective			
Baseline costs	2330	2807	− 477
Out-of-pocket expenses	337	313	24
Emergency costs	282	1171	− 890
Diagnosis and treatment	272	1056	− 783
Pharmaceutical service (including medication)	9	116	− 106
Outpatient costs	4826	4106	721
Diagnosis	498	509	− 12
Treatment	1058	1201	− 143
Pharmaceutical service	83	80	4
Medication	3187	2316	871
Inpatient costs	1322	2153	− 831
Room	270	457	− 187
Diagnosis	87	141	− 54
Therapy and examination	612	1023	− 412
Pharmaceutical service	20	31	− 11
Medication	164	282	− 117
Special materials	169	220	− 51
Pharmacy costs	876	696	180
Pharmaceutical service	28	29	− 1
Medication	829	636	193
Special materials	19	31	− 12
Healthcare sector costs	7306	8126	− 820
Healthcare sector costs (adjusted^a^)	8374	8716	− 342

ΔCost: difference in costs per subject between GLP-1RA and insulin users over a mean follow-up period; GLP-1RAs: glucagon-like peptide-1 receptor agonists

All costs are presented in 2019 US dollars and rounded to the nearest integer

^a^Costs were measured for the follow-up period from the first prescription of the stable use of a GLP-1RA or insulin (the index date) to withdrawal from Taiwan’s National Health Insurance program, death, or the end of 2016, whichever came first. The total medical costs were adjusted for the baseline medical costs, which were the total medical costs within 1 year before the index date

### Base-case and sensitivity analyses

In Table 3, using three times Taiwan’s GDP as the WTP threshold (US$77,679), the base-case analysis indicated that using a GLP-1RA versus insulin was cost-effective for preventing one case with all-cause mortality or hospitalized hypoglycemia from the payer perspective, and cost-saving from the healthcare sector perspective. That is, compared to insulin, 57 and 30 patients would need to be treated with a GLP-1RA for a mean of 2.3 years to prevent one case of all-cause mortality and hospitalized hypoglycemia, respectively, which would cost US$54,851 and US$29,115 per case of all-cause mortality and hospitalized hypoglycemia prevented, respectively, from the payer perspective, and would save US$19,391 and US$10,293, respectively, from the healthcare sector perspective.

**Table 3:** Results of cost-effectiveness analysis of GLP-1RAs versus insulin (base-case and sensitivity analyses)

	ΔEffectiveness^a^	ΔCost^b^		Cost per case of event prevented (ICER)	
	NNT^c^	Third-party payer perspective	Healthcare sector perspective	Third-party payer perspective	Healthcare sector perspective
Base-case analysis (ITT scenario for effectiveness estimation + cost measured from the index date until the end of observation)					
All-cause death	56.63	969	− 342	54,851	− 19,391
Hospitalized hypoglycemia	30.06	969	− 342	29,115	− 10,293
1st sensitivity analysis (ITT scenario for effectiveness estimation + cost measured from the index date until the occurrence of study event)					
All-cause death	56.63	971	− 325	55,002	− 18,425
Hospitalized hypoglycemia	30.06	1128	47	33,901	1399
2nd sensitivity analysis (AT scenario for effectiveness estimation + cost measured from the index date until the end of observation)					
All-cause death	100.80	969	− 342	97,633	− 34,515
Hospitalized hypoglycemia	29.79	969	− 342	28,856	− 10,201
3rd sensitivity analysis (AT scenario for effectiveness estimation + cost measured from the index date until the occurrence of study event)					
All-cause death	100.80	971	− 325	97,901	− 32,796
Hospitalized hypoglycemia	29.79	1128	47	33,600	1387

ΔCost: difference in costs per subject between GLP-1RA and insulin users over a mean follow-up period; GLP-1RAs: glucagon-like peptide-1 receptor agonists; ICER: incremental cost-effectiveness ratio; ITT: intention-to-treat; AT: as-treated

^a^In the primary and 1st sensitivity analyses, the follow-up period of effectiveness estimation was measured from the first prescription of the stable use of a GLP-1RA or insulin (the index date) to withdrawal from Taiwan’s National Health Insurance program, event occurred, death, or the end of 2016, whichever came first (i.e., ITT analyses). In the 2nd and 3rd sensitivity analyses, the follow-up period of effectiveness estimation was measured from the first prescription of the stable use of a GLP-1RA or insulin (the index date) to the occurrence of a study event of interest (i.e., all-cause death or hospitalized hypoglycemia), withdrawal from Taiwan’s National Health Insurance program, death, discontinuation of the current treatment (i.e., GLP-1RA or insulin), or the end of 2016, whichever cam first (i.e., AT analyses)

^b^In the primary and 2nd sensitivity analyses, costs were measured for the follow-up period from the first prescription of the stable use of a GLP-1RA or insulin (the index date) to withdrawal from Taiwan’s National Health Insurance program, death, or the end of 2016, whichever came first. In the 1st and 3rd sensitivity analyses, costs were measured for the follow-up period from the first prescription of the stable use of a GLP-1RA or insulin (the index date) to the occurrence of a study event of interest (i.e., all-cause death or hospitalized hypoglycemia), withdrawal from Taiwan’s National Health Insurance program, death, or the end of 2016, whichever came first

^c^The NNT estimate was calculated based on the survival probabilities from the Cox proportional hazard model with the adjustment of the imbalanced baseline patient characteristics shown in Additional file 1: Table S2

All costs are presented in 2019 US dollars and rounded to the nearest integer

Consistent with the base-case analysis, sensitivity analyses demonstrated that using GLP-1RAs versus insulin was either cost-saving or cost-effective, except for the analyses where the treatment effectiveness was estimated based on the AT scenario in the second and third sensitivity analyses, showing that a GLP-1RA versus insulin therapy was not cost-effective for averting one case of all-cause death from the payer perspective. The nonparametric bootstrapping analysis showed that the 95% CIs for ICERs were in a range of − US$54,391 to US$231,475 (− US$70,894 to $37,945) for all-cause death and − US$28,870 to US$122,865 (− S$37,630 to US$20,141) for hospitalized hypoglycemia from the payer perspective (healthcare sector perspective). Additional file 1: Fig. S4 shows the cost-effectiveness acceptability curves; based on three times Taiwan’s GDP, the probabilities of using a GLP-1RA versus insulin therapy being cost-effective for preventing one case of all-cause mortality and hospitalized hypoglycemia were 60% (100%) and 80% (100%), respectively, from the payer perspective (healthcare sector perspective).

### Systematic review

A total of 21 CEAs (including the present study) were identified in the systematic review (Additional file 1: Fig. S3, Table S5), with the majority of studies focusing on a specific GLP-1RA drug, including 12 on exenatide, 2 on lixisenatide, 3 on dulaglutide, 1 on albiglutide, and 1 on semaglutide; two studies did not focus on a specific GLP-1RA drug. All studies adopted the model-based simulation approach using data predominately derived from clinical trials, except for our study, which employed a trial/study-based analysis approach using real-world effectiveness and cost data, to conduct CEA. Across these CEAs, relative to insulin, the use of a GLP-1RA could be dominated or had an ICER of − US$22,682 to US$90,646 per QALY gained from the payer perspective, an ICER of US$1982 to US$42,679 per QALY gained from the healthcare sector perspective, and an ICER of − US$21,488 to US$28,490 per QALY gained from the societal perspective. Detailed descriptions of these CEAs are provided in Additional file 1: Table S5.

**Fig. 1:**
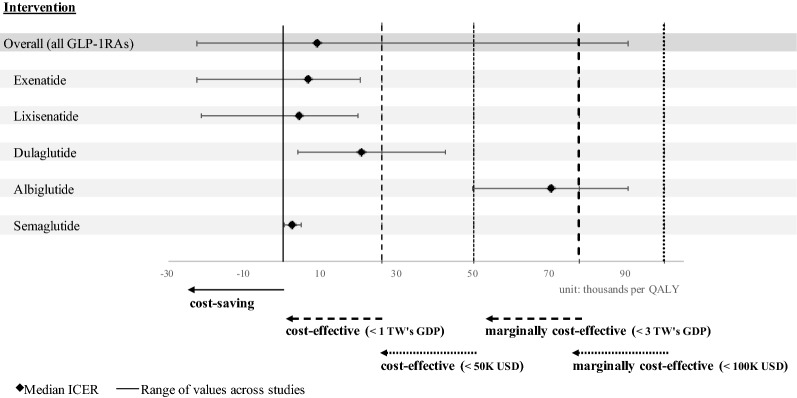
Summary of existing studies on cost-effectiveness of glucagon-like peptide-1 receptor agonists (GLP-1RAs) versus insulin therapy. GLP-1RAs: glucagon-like peptide-1 receptor agonists; TW: Taiwan; GDP: gross domestic product; USD: United States dollars; ICER: incremental cost-effectiveness ratio; QALY: quality-adjusted life year. (1) In 2019, the one-time per capita Taiwan’s GDP was US$25,893 and the three-time per capita Taiwan’s GDP was US$77,679. (2) This figure includes the articles summarized in Additional file 1: Table S5 expect for two published studies (Edwards et al. 2006 and Woehl et al. 2008) and our study. This is because the cost per QALY was not reported in Edwards et al.’s study (2006) and our study, and the use of exenatide was dominated by insulin glargine in Woehl et al.’s study (2008)

Figure 1 shows the systematic review findings. Overall, using a GLP-1RA versus insulin was cost-effective. Exenatide, liraglutide, lixisenatide, dulaglutide, and semaglutide were cost-effective given a WTP threshold of US$50,000 per QALY gained, and albiglutide was marginally cost-effective given a WTP threshold of US$100,000 per QALY gained.

## Discussion

To our knowledge, this is the first real-world-study-based CEA of using GLP-1RAs versus insulin for T2D patients who require a greater glucose-lowering effect of injection therapy, and the first systematic review on this topic. Our findings suggest that compared to insulin therapy, the higher drug acquisition, pharmacy, and outpatient costs associated with the real-world use of a GLP-1RA is offset by a reduction in healthcare-related expenses associated with emergency room visits and inpatient admissions owing to reduced risks of all-cause mortality and hospitalized hypoglycemia. The use of a GLP-1RA versus insulin therapy in routine clinical practice is thus cost-effective and cost-saving from the perspectives of a third-party payer and the healthcare sector, respectively.

### Comparison of present study with previous studies and clinical and policy implications of present study

A direct comparison of the present study with the studies included in our systematic review would be a challenge due to the use of different analytic approaches, cost-effectiveness metrics, analysis perspectives, and healthcare settings. Nevertheless, the present study-based CEA provides supporting data for the favorable economic outcomes of the real-world use of a GLP-1RA versus insulin among T2D patients who require intensified injection therapy. Consistently, a recent systematic review and meta-analysis of GLP-1RAs versus other GLAs showed that the use of GLP-1RAs is cost-effective compared to insulin therapy [46]. However, caution should be given to this review because most studies were from high-income countries and insulin degludec/liraglutide (IDegLira) was included in the GLP-1RA group.

From the perspective of the clinical care of T2D patients who require intensified injection therapy, our findings are consistent with existing treatment guidelines [1, 3] and a real-world study [47], showing that using a GLP-1RA versus insulin therapy has therapeutic benefits in terms of reduced risks of hypoglycemia and all-cause death. Our real-world evidence of the comparative effectiveness of GLP-1RAs versus insulin complements existing trial evidence by translating the efficacy of GLP-1RAs in clinical trials to its effectiveness in clinical practice among a broader spectrum of real-world patient populations for supporting the rational selection of GLAs in clinical practice [48].

From the perspective of healthcare reimbursement by a third-party payer, the results of our CEA and systematic review suggest that the use of GLP-1RAs versus insulin is cost-effective for T2D patients who require intensified injection therapy. Our study demonstrated that compared to insulin therapy, the higher drug acquisition, pharmacy, and outpatient costs associated with the use of GLP-1RAs are offset by reduced emergency room and inpatient costs. This is supported by recent studies from US and German adults with T2D, which reported that the use of GLP-1RAs versus insulin resulted in lower emergency room visits and inpatient admissions but higher drug acquisition costs after treatment initiation [49, 50]. Therefore, evidence of the comparative cost-effectiveness of GLP-1RAs versus insulin therapy from the present study could support the formulation of healthcare reimbursement policies in real-world clinical practice.

### Methodological strengths of present study for supporting analytic framework of future real-world CEAs

The present CEA has several methodological strengths. First, for generalizability to real-world practice, we identified a real-world representative cohort of T2D patients who were GLP-1RA and insulin users in the analyses and implemented a rigorous matching algorithm to match on a range of baseline patient characteristics (demographics, comorbidities, diabetes severity, concurrent medications) to achieve a greater level of between-group comparability. Second, for the validity of study results, we adopted sophisticated analytic procedures to estimate the treatment effectiveness and healthcare costs, including NNT measures derived from the survival analysis with adjustment for imbalanced baseline characteristics between study groups, and downstream healthcare costs estimated from a regression analysis with adjustment for baseline costs. Third, efforts were made to minimize potential bias due to heterogenous medication adherence in real-world patients, namely (1) the study cohort was restricted to include only stable users of study drugs to eliminate the potential confounding effect from the short-term use or non-adherence of study drugs and (2) sensitivity analyses were performed based on the AT scenario to estimate treatment effectiveness, with patients who discontinued or switched study drugs censored to corroborate our base-case analysis results based on the ITT scenario. Lastly, a series of sensitivity analyses were conducted to corroborate the base-case analysis results and examine the validity of our findings.

From the perspective of scientific research, existing recommendations for analytic procedures in real-world-study-based CEAs remain insufficient [7, 8, 51]. Our analytic procedures for conducting a real-world-study-based CEA, which carefully consider study cohort comparability, the validity of effectiveness and cost parameter estimations, and the uncertainty of study data, results, and assumptions, provide a methodological framework for future real-world-study-based CEAs. This analytic framework can facilitate the translation of cost-effectiveness evidence from real-world comparative effectiveness research to inform healthcare and policy decision-making. Moreover, a real-world-study-based CEA is an analytic framework for health economic evaluation that complements evidence from trial- or model-based CEAs because it is more generalizable; that is, it reflects the heterogeneity of population characteristics, treatment effects, and resource utilization and costs in real-world practice settings.

### Study limitations

Several limitations of our study need to be addressed. First, GLP-1RAs are a relatively new class of GLA, and thus real-world experiences and patient outcomes of using this class of drug may be limited in Taiwan. Considering the chronic nature of diabetes-related complications (e.g., CVDs), the follow-up period of this real-world study may not be sufficient to assess the long-term benefits of GLP-1RA therapy. Second, due to data unavailability, potential unmeasured residual confounding such as physicians’ preference or behavior and patients’ laboratory data may not have been eliminated in the estimation of treatment effectiveness or costs. However, our rigorous matching procedures with the intention to achieve a greater level of between-group comparability on a wide range of baseline patient characteristics (i.e., prior GLA exposure history, comorbidities, diabetes severity, and co-medications) and the regression analyses with adjustment for imbalanced baseline characteristics may have minimized this concern. Third, our analysis did not include costs from the informal healthcare sector (e.g., transportation costs) and from the non-healthcare sector (e.g., lost productivity for individuals) due to data unavailability, and thus the results of this study may not be extrapolated to a societal perspective. Fourth, adverse treatment effects and diabetes-related complications could greatly reduce a person’s quality of life and thus should be considered in a cost-utility analysis. Due to a lack of representative utility data for Taiwanese patients with T2D, a cost-utility analysis was not conducted. However, the reduced risks of hospitalized hypoglycemia and all-cause mortality associated with the use of a GLP-1RA versus insulin may lead to a favorable cost-utility profile for using a GLP-1RA. Fifth, due to limited numbers of study subjects in different individual GLP-1RA and insulin therapies, further assessment of comparative effectiveness and cost-effectiveness of treatments by the more granular level of different individual drugs would be underpowered. Moreover, given no heterogeneous treatment effects found by age, gender, or diabetes severity for GLP-1RAs versus insulin in our previous analyses [6] and limited study subjects with different patient characteristics in the present study, performing the cost-effectiveness analysis stratified by different patient demographic or clinical characteristics would be infeasible. Therefore, future research with sufficient study subjects that can be classified by different individual drugs and patient characteristics is encouraged, which can provide more individualized and precise suggestions on treatment selections. Lastly, the generalizability of the present CEA may be limited to healthcare systems with a universal health insurance coverage.

## Conclusions

This real-world health economic evaluation along with a comprehensive systematic review suggest that the use of a GLP-1RA versus insulin has a high likelihood of being cost-effective for T2D patients who require intensified injection therapy from a third-party payer, healthcare sector, and societal perspectives. This study not only provides real-world cost-effectiveness findings that complement evidence from trial- or model-based CEAs but also summarizes up-to-date cost-effectiveness evidence, both of which are of importance for clinical decision-making regarding treatment selections and the formulation of healthcare reimbursement policies in real-world practice. Future research can apply our analytic framework to perform a real-world-study-based CEA for either corroborating our findings or translating more real-world comparative effectiveness research to inform clinical professionals and health policymakers when prioritizing treatment strategies for patients with T2D given limited healthcare resources.

## Supplementary information

**Additional file 1: Table S1.** Impact inventory for components considered in the cost-effectiveness analysis. **Fig. S1.** Framework of study design for cost effectiveness analysis. **Table S2.** CHEERS Checklist. **Fig. S2.** Flowchart of selection of study cohort patients. **Table S3.** Baseline characteristics of study patients before and after three-step matching. **Table S4.** Definitions of study outcomes of interest. **Fig. S3.** Flowchart for articles included in the systematic review. **Fig. S4.** Cost-effectiveness acceptability curve. **Table S5.** Description of existing studies on cost-effectiveness of glucagon-like peptide-1 receptor agonists (GLP-1RAs) versus insulin therapy.

## Data Availability

Data sharing is not applicable to this study as data management and analysis were only allowed to be conducted in Health and Welfare Data Science Center in Taiwan for data privacy and safety concerns.

## Abbreviations

AT: As-treated
CEA: Cost-effectiveness analysis
CPI: Consumer price index
CVD: Cardiovascular disease
GDP: Gross domestic product
GLA: Glucose-lowering agent
GLP-1RA: GLP-1 receptor agonist
HR: Hazard ratio
ICER: Incremental cost-effectiveness ratio
ITT: Intention-to-treated
MACE: Major adverse cardiovascular event
NHI: National Health Insurance
NHIRD: National Health Insurance Research Database
NNT: Number needed to treat
OOP: Out-of-pocket
QALY: Quality-adjusted life year
T2D: Type 2 diabetes
WTP: Willingness-to-pay
