# Supplementary material for: Cost-effectiveness of GLP-1 receptor agonists versus insulin for the treatment of type 2 diabetes: a real-world study and systematic review
Source: Cardiovasc Diabetol. 2021 Jan 19;20:21. doi: 10.1186/s12933-020-01211-4 (PMC7816439; doi:10.1186/s12933-020-01211-4)
Supplement: Supplementary file 1 — Additional file 1: Table S1. Impact inventory for components considered in the cost-effectiveness analysis. Fig. S1. Framework of study design for cost effectiveness analysis. Table S2. CHEERS Checklist. Fig. S2. Flowchart of selection of study cohort patients. Table S3. Baseline characteristics of study patients before and after three-step matching. Table S4. Definitions of study outcomes of interest. Fig. S3. Flowchart for articles included in the systematic review. Fig. S4. Cost-effectiveness acceptability curve. Table S5. Description of existing studies on cost-effectiveness of glucagon-like peptide-1 receptor agonists (GLP-1RAs) versus insulin therapy. [file 12933_2020_1211_MOESM1_ESM.docx]

**Table S1: Impact inventory for components considered in the cost-effectiveness analysis**

| Sector | Type of impact | Perspective | | Note on sources of evidence |
| --- | --- | --- | --- | --- |
|  | (list category within each sector with unit of measure if relevant) | Third-party payer | Healthcare sector |  |
| Formal Healthcare Sector | | | | |
| Health | Health outcomes (effects) |  |  |  |
|  | Longevity effects, years | ✓ | ✓ |  |
|  | Health-related quality of life effects, QALY | 🗶 | 🗶 | Data unavailable |
|  | NNT to prevent hospitalized hypoglycemia | ✓ | ✓ | Converted from the survival probabilities derived from Cox proportional model analyses (Altman et al. 1999) |
|  | NNT to prevent all-cause death | ✓ | ✓ |  |
|  | Medical costs, $ |  |  |  |
|  | Paid for by third-party payers | ✓ | ✓ | Retrieved from NHIRD |
|  | Paid for by patients out-of-pocket | 🗶 | ✓ |  |
|  | Future-related medical costs (payers and patients) | ✓ | ✓ |  |
|  | Future-unrelated medical costs (payers and patients) | ✓ | ✓ |  |

Abbreviations: QALY, quality-adjusted life year; NNT, number needed to treat; NHIRD, National Health Insurance Research Database.

**Fig. S1: Framework of study design for cost effectiveness analysis**

- **Comparable** study groups achieved by **3-step matching** on:

1. **Initiation date (index date) of study drugs** to align the cohort entry time between study groups
2. **Patterns of prior glucose-lowering agent use** measured by medication procession ratios to equalize the past exposure of glucose-lowering agents between study groups
3. **Potential confounders** (i.e., demographics, comorbidities, diabetes severity, CVD-related co-medications) measured by propensity scores to balance baseline patient characteristics between study groups

**Cohort for CEA:**
comparable study groups

(GLP-1RAs vs. insulin)

**Effectiveness parameter for CEA**

- **NNT** measured based on 1) incidence rates and 2) Cox model-estimated survival probabilities
- **Perspectives** for cost estimation: 1) healthcare sector and 2) third-party payer (i.e., NHIA)
- Costs adjusted for **imbalanced baseline costs** between study groups

**Cost parameter for CEA**

**CEA: base-case analysis**based on ITT scenario for effectiveness estimation + cost measured from the index date until the end of observation

**CEA: 3 sensitivity analyses based on different estimations of effectiveness and cost**1) ITT scenario for effectiveness estimation + cost measured from the index date until the occurrence of study event

2) AT scenario for effectiveness estimation + cost measured from the index date until the end of observation

3) AT scenario for effectiveness estimation + cost measured from the index date until the occurrence of study event

Abbreviations: GLP-1RAs, glucagon-like peptide-1 receptor agonists; NNT, number needed to treat; NHIA, National Health Insurance Administration; CEA, cost-effectiveness analysis; CVD, cardiovascular disease; ITT, intention-to-treat; AT, as-treated.

**Table S2: CHEERS Checklist**

| Section/item | Item no. | Recommendation | Page No./Line No. |
| --- | --- | --- | --- |
| **Title and abstract** |  |  |  |
| Title | 1 | Identify the study as an economic evaluation or use more specific terms such as “cost-effectiveness analysis”, and describe the interventions compared. | 1 |
| Abstract | 2 | Provide a structured summary of objectives, perspective, setting, methods (including study design and inputs), results (including base case and uncertainty analyses), and conclusions. | 2/1-3/1 |
| **Introduction** |  |  |  |
| Background and objectives | 3 | Provide an explicit statement of the broader context for the study. Present the study question and its relevance for health policy or practice decisions. | 4/1-5/16 |
| **Methods** |  |  |  |
| Target population and subgroups | 4 | Describe characteristics of the base case population and subgroups analysed, including why they were chosen. | 6/16-7/8, Supplementary Figure 2 |
| Setting and location | 5 | State relevant aspects of the system(s) in which the decision(s) need(s) to be made. | 6/9-15 |
| Study perspective | 6 | Describe the perspective of the study and relate this to the costs being evaluated. | 8/10-9/5, Table 2 |
| Comparators | 7 | Describe the interventions or strategies being compared and state why they were chosen. | 4/4-21, 6/17-21 |
| Time horizon | 8 | State the time horizon(s) over which costs and consequences are being evaluated and say why appropriate. | 8/10-10/6 |
| Discount rate | 9 | Report the choice of discount rate(s) used for costs and outcomes and say why appropriate. | N/A |
| Choice of health outcomes | 10 | Describe what outcomes were used as the measure(s) of benefit in the evaluation and their relevance for the type of analysis performed. | 7/10-8/8, Supplementary Table 4 |
| Measurement of effectiveness | 11a | Single study-based estimates: Describe fully the design features of the single effectiveness study and why the single study was a sufficient source of clinical effectiveness data. | 6/9-15, 7/10-8/8 |
| Measurement and valuation of preference based outcomes | 12 | If applicable, describe the population and methods used to elicit preferences for outcomes. | N/A |
| Estimating resources and costs | 13a | Single study-based economic evaluation: Describe approaches used to estimate resource use associated with the alternative interventions. Describe primary or secondary research methods for valuing each resource item in terms of its unit cost. Describe any adjustments made to approximate to opportunity costs. | 6/16-23, 9/18-10/17 |
| Currency, price date and conversion | 14 | Report the dates of the estimated resource quantities and unit costs. Describe methods for adjusting estimated unit costs to the year of reported costs if necessary. Describe methods for converting costs into a common currency base and the exchange rate. | 8/20-23 |
| Choice of model | 15 | Describe and give reasons for the specific type of decision-analytical model used. Providing a figure to show model structure is strongly recommended. | N/A |
| Assumptions | 16 | Describe all structural or other assumptions underpinning the decision-analytical model. | N/A |
| Analytical methods | 17 | Describe all analytical methods supporting the evaluation. This could include methods for dealing with skewed, missing, or censored data; extrapolation methods; methods for pooling data; approaches to validate or make adjustments (such as half cycle corrections) to a model; and methods for handling population heterogeneity and uncertainty. | 7/1-8, 7/22-8/8, 8/18-9/5 |
| **Results** |  |  |  |
| Study parameters | 18 | Report the values, ranges, references, and, if used, probability distributions for all parameters. Report reasons or sources for distributions used to represent uncertainty where appropriate. Providing a table to show the input values is strongly recommended. | Table 1, 2, Supplementary Figure 1 |
| Incremental costs and outcomes | 19 | For each intervention, report mean values for the main categories of estimated costs and outcomes of interest, as well as mean differences between the comparator groups. If applicable, report incremental cost-effectiveness ratios. | Table 3 |
| Characterising uncertainty | 20a | Single study-based economic evaluation: Describe the effects of sampling uncertainty for the estimated incremental cost and incremental effectiveness parameters, together with the impact of methodological assumptions (such as discount rate, study perspective). | Table 3, Supplementary Figure 1 |
| Characterising heterogeneity | 21 | If applicable, report differences in costs, outcomes, or cost-effectiveness that can be explained by variations between subgroups of patients with different baseline characteristics or other observed variability in effects that are not reducible by more information/ | N/A |
| **Discussion** |  |  |  |
| Study findings, limitations, generalizability, and current knowledge | 22 | Summarize key study findings and describe how they support the conclusions reached. Discuss limitations and the generalizability of the findings and how the findings fit with current knowledge. | 14/5-14, 15/21-17/5, 17/7-18/6 |
| **Other** |  |  |  |
| Source of funding | 23 | Describe how the study was funded and the role of the funder in the identification, design, conduct, and reporting of the analysis. Describe other non-monetary sources of support. | 19/21-25 |
| Conflicts of interest | 24 | Describe any potential for conflict of interest of study contributors in accordance with journal policy. In the absence of a journal policy, we recommend authors comply with International Committee of Medical Journal Editors recommendations. | 20/9-10 |

**Fig. S2: Flowchart of selection of study cohort patients**

All T2D patients during 2008-2015 (N=1,853,298)

Exclusion

- Aged < 18 years at T2D diagnosis (N=9,356)
- Gender undefined (N=10,438)

Adult patients with T2D diagnosis (N=1,834,563)

T2D patients exposed to a GLP-1RA (N=4,191) or insulin (N=204,146) therapy during 2011-2015

Exclusion

- Unstable users of study drugs (a GLP-1RA or insulin) (N=128,533)

Target patients with stable use sets of a GLP-1RA or insulin

**Insulin group**

Before matching:

(N=108,745 subjects)

(n=2,398,584 stable sets)

**GLP-1RA group**

Before matching: (N=3,435 subjects)

(n=34,463 stable sets)

Abbreviations: T2D, type 2 diabetes; GLP-1RAs, glucagon-like peptide-1 receptor agonists.

**Notes for study cohort identification:**

Patients with type 2 diabetes (T2D) were defined based on the International Classification of Diseases, Ninth Revision, Clinical Modification (ICD-9-CM) codes (250.x0, 250.x2, x=0-9) within a year for T2D in at least: 1) two outpatient visits, (2) one inpatient visit, or (3) one outpatient visit with any glucose-lowering agent (GLA) prescriptions. We further identified patients with newly diagnosed T2D during 2008-2015 as those without T2D diagnosis in the preceding year. Because the reimbursement for glucagon-like peptide-1 receptor agonists (GLP-1RAs) in Taiwan’s National Health Insurance program was not available until 2011, we identified the users of GLP-1RAs or insulin therapy during 2011-2015 where the medication ingredients were defined according to the Anatomical Therapeutic Chemical Classification System. Moreover, to exclude temporary treatment effects due to short-term drug use, only the stable users in the study groups (a GLP-1RA and insulin) were included in the analyses. A stable user was defined as a patient with stable use sets. A stable use set was defined as at least three drug refills with any gap between two consecutive prescriptions of less than 30 days. A stable user of a GLP-1RA or insulin can have multiple stable use sets of that drug chronologically over time. For subjects in the GLP-1RA group, the first prescription date of the stable use of a GLP-1RA was defined as the index date, and for subjects in the insulin group, the first prescription date of a stable use set of insulin was defined as the index date. Detailed definitions of stable users and index dates can be found elsewhere [1]. The follow-up period for each patient was from the index date until death, loss to follow-up, development of a clinical outcome of interest, or the end of 2016, whichever came first (based on the intention-to-treated analysis scenario). Furthermore, the comparability of baseline patient characteristics between two study groups was achieved by a three-step matching algorithm to match on 1) the index date, 2) prior exposure of GLA prescriptions in the year before the index date, and 3) patient demographic and clinical characteristics (e.g., diabetes severity, comorbidities, co-medications for cardiovascular diseases) using the propensity score matching approach. To include as many GLP-1RA users as possible in the analyses and match them to the most comparable insulin users with similar baseline patient characteristics, we allowed the stable use sets of insulin for the subjects to be re-used through the matching process. Details of the three-step matching algorithm are described elsewhere [31].

**Table S3: Baseline characteristics of study patients before and after three-step matching**

| **Characteristics**† | **Before matching** | |  | **After matching** | |
| --- | --- | --- | --- | --- | --- |
|  | **GLP-1RAs** | **insulin** |  | **GLP-1RAs** | **insulin** |
| **Number of subjects**‡ | 3,435 | 2,398,584 |  | 1,022 | 1,022 |
| **Demographics at the index date** |  |  |  |  |  |
| Age (years, mean ± SD) | 46.15±11.91* | 55.5±16.98 |  | 47.18±12.18* | 51.57±14.13 |
| Male (%) | 48.03 | 54.59 |  | 46.87 | 54.70 |
| **Diabetes duration** (years, mean ± SD) | 3.69±1.98* | 3.69±2.08 |  | 3.67±2.05 | 4.12±1.97 |
| **Comorbidity history (%)** |  |  |  |  |  |
| Hypertension | 57.7* | 55.64 |  | 54.11 | 57.73 |
| Hyperlipidemia | 69.58* | 51.23 |  | 66.73* | 68.10 |
| TIA or stroke | 4.22* | 12.38 |  | 5.38 | 5.48 |
| Heart failure | 3.11 | 6.66 |  | 4.01 | 3.33 |
| Myocardial infarction | 1.51 | 2.27 |  | 1.86 | 1.17 |
| Ischemic heart diseases | 10.86 | 15.68 |  | 12.72 | 11.15 |
| **CIC category (%)** | 13.43 |  |  |  |  |
| Cancers | 3.78* | 7.93 |  | 5.77 | 4.50 |
| Gastrointestinal diseases | 24.69 | 27.56 |  | 27.30 | 26.03 |
| Musculoskeletal diseases | 31.67* | 30.92 |  | 31.12 | 33.07 |
| Pulmonary diseases | 7.71 | 10.16 |  | 6.16 | 8.02 |
| Substance abuse complexity | 2.47 | 4.28 |  | 3.62 | 2.54 |
| Mental illnesses | 9.96 | 10.79 |  | 11.06 | 10.76 |
| **Prior diabetes-related complications (%)** |  |  |  |  |  |
| Retinopathy | 15.31* | 21.46 |  | 19.67 | 18.59 |
| Nephropathy | 25.07* | 35.61 |  | 26.61 | 27.10 |
| Neuropathy | 12.58 | 16.76 |  | 16.34* | 15.36 |
| Peripheral vascular diseases | 3.90 | 7.21 |  | 4.31 | 5.77 |
| Cerebrovascular diseases | 3.38* | 10.74 |  | 4.11 | 4.50 |
| Cardiovascular diseases | 13.62 | 20.79 |  | 15.56 | 13.99 |
| Metabolic complications | 2.56 | 8.68 |  | 5.28 | 2.64 |
| **Past use of GLAs** (MPR, mean ± SD) |  |  |  |  |  |
| Metformin | 0.45±0.42* | 0.26±0.40 |  | 0.41±0.42 | 0.40±0.40 |
| Sulfonylurea | 0.37±0.44* | 0.20±0.36 |  | 0.29±0.40 | 0.29±0.39 |
| Meglitinide | 0.05±0.20* | 0.06±0.21 |  | 0.05±0.20 | 0.05±0.19 |
| Thiazolidinedione | 0.11±0.26* | 0.05±0.18 |  | 0.05±0.19 | 0.05±0.19 |
| Acarbose | 0.12±0.27* | 0.09±0.25 |  | 0.08±0.23 | 0.08±0.23 |
| DPP-4i | 0.35±0.41* | 0.17±0.34 |  | 0.26±0.38 | 0.25±0.37 |
| Insulin | 0.25±0.40* | 0.83±0.45 |  | 0.63±0.42 | 0.61±0.36 |
| **Past use of CVD-related drugs (%)** |  |  |  |  |  |
| Lipid-modifying agents | 62.59* | 45.99 |  | 58.41 | 59.20 |
| α-blockers | 3.38 | 5.06 |  | 3.62 | 3.33 |
| β-blockers | 26.87 | 27.51 |  | 27.59 | 24.27 |
| Agents acting on the renin-angiotensin system | 36.16 | 31.80 |  | 35.71 | 35.52 |
| Diuretics | 14.73 | 22.00 |  | 17.51 | 15.75 |
| CCB | 25.74 | 30.68* |  | 29.55 | 26.13 |
| Antiarrhythmics | 1.11 | 2.21 |  | 1.08 | 1.27 |
| Cardiac glycosides | 1.02 | 2.25 |  | 1.57 | 1.27 |
| Vasodilators used in cardiac disease | 6.35 | 12.11 |  | 8.51 | 8.41 |
| Anti-platelets | 21.60 | 33.13 |  | 24.85 | 23.68 |
| Anti-coagulants | 0.82 | 1.77 |  | 1.27 | 0.49 |

Abbreviations: GLP-1RAs, glucagon-like peptide-1 receptor agonists; SD, standard deviation; TIA, transient ischemic attack; CIC, chronic illness with complexity; GLAs, glucose-lowering agents; MPR, medication possession ratio; DPP-4i, dipeptidyl peptidase-4 inhibitor; CVD, cardiovascular disease; CCB, calcium channel blockers.

^*^ A significant difference in baseline patient characteristics was found between GLP-1RA and insulin groups, as indicated by absolute standardized mean difference (SMD) > 0.1.

^†^ All patient characteristics were measured in the year prior to the index date (i.e., the first date of the stable use of a GLP-1RA or the first date of a stable use set of insulin), except for age and gender, which were measured at the index date. All characteristics, except the past utilization of GLAs, were included in the estimation of propensity scores for the study cohort matching.

^‡^ The number of subjects for the GLP-1RA group was the number of stable users, whereas that for the insulin group was the number of stable use sets.

**Table S4: Definitions of study outcomes of interest**

| Outcome | ICD-9-CM disease code | ICD-10-CM disease code |
| --- | --- | --- |
| Cardiovascular disease^a-i^ | | |
| Acute myocardial infarction | 410 | I21 |
| Ischemic heart disease | 411, 413, 414, V45.81, V45.82 | I25 |
| Heart failure | 428 | I50 |
| Stroke | 430-437, V12.54 | I61, I62 |
| Cardiogenic shock | 785.51 | R57 |
| Sudden cardiac arrest | V12.53 | Z86.74 |
| Arteriosclerotic cardiovascular disease | 429.2 | I25.10 |
| Arrhythmia | 426, 427 | I44, I45, I46, I47, I48, I49 |
| Hypoglycemia^f, j, k^ | 251.0, 251.1, 251.2, 270.3, 962.3 | E15, E160, E161, E162, E11.64, T38.3 (except T38.3X6) |
| All-cause death | Directly linked to the records of cause of death statistics from Ministry of Health and Welfare | |

Notes:

1. Cardiovascular disease (CVD) was a composite outcome that included acute myocardial infarction, stroke, heart failure, ischemic heart disease, arrhythmia, arteriosclerotic cardiovascular disease, cardiogenic shock, and sudden cardiac arrest.

2. Fatal CVD was a death due to any cardiovascular disease.

3. Three-point major adverse cardiovascular events (MACE) included non-fatal acute myocardial infarction, non-fatal stroke, and fatal CVD.

a. *Am J Manag Care.* 2012;18(11):721-6.

b. *Nutr, Metab Cardiovasc Dis.* 2014;24(1):10-7.

c. *Cardiovasc Diabetol.* 2014;13:3.

d. *Circulation*. 2002;106(13):1634-9.

e. *Crit Care*. 2015;19:452.

f. *J Am Heart Assoc*. 2016;5(1):e002667.

g. *J. Clin Epidemiol.* 2002;55(6):602-7.

h. *BMJ*. 2016;352:i843.

i. *Am. Heart J.* 2010;160(2):264-271.e1.

j. *BMC Endocr Disord*. 2008;8:4.

k. *Diabetes Care*. 2019;42(4):e58-e59.

**Fig. S3: Flowchart for articles included in the systematic review**

All available studies on the cost-effectiveness of glucagon-like peptide-1 receptor agonists versus insulin were retrieved from MEDLINE and EMBASE between the inception of the databases and the end of August 2020. The search strategy, based on the framework of “Patient Intervention Comparison Outcome (PICO)”, was: **P**: type 2 diabetes; **I**: glucagon-like peptide-1 receptor agonist (including synonyms); **C**: insulin (including synonyms); **O**: cost-effectiveness analyses, cost-utility analyses, cost-benefit analyses, cost per additional quality-adjusted life year (QALY) gained, cost per additional life year gained (LYG), or cost per additional disability adjusted life year averted. 120 and 357 articles were identified from MEDLINE and EMBASE, respectively. After the exclusion of a total of 434 articles due to duplicates, unmatched PICO, conference abstracts, and unavailable full-text articles, a total of 20 studies were included in the final review. The details of inclusion/exclusion criteria were outlined in the flowchart below.

Potentially relevant articles from electronic databases^*^: 477

MEDLINE: 120

EMBASE: 357

Titles or abstracts that did not meet PICO criteria: 434^*^

Article retrieved for more detailed evaluation: 43

MEDLINE: 21

EMBASE: 22

Records after duplicates removed: 21

Full-text articles are not available or not in English: 2

Number of studies in systematic review: 20^†^

^*^ This exclusion included seven studies that compared IDegLira versus insulin. They were excluded because IDegLira, a combination of liraglutide and insulin degludec, did not meet our PICO criteria, where “I” referred to glucagon-like peptide-1 receptor agonists only.

^†^ There was only one article that conducted a cost-effectiveness analysis in which the QALY outcome was not analyzed.

**Fig. S4: Cost-effectiveness acceptability curve**

1. **All-cause death**

WTP: US$77,679

**
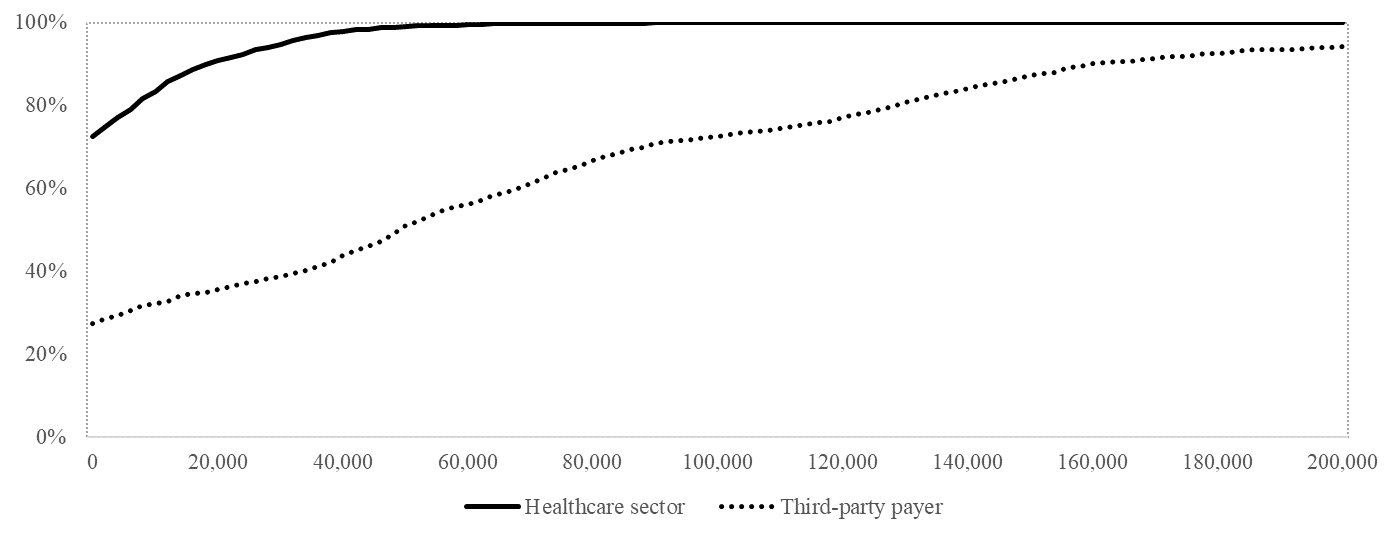
**

1. **Hospitalized hypoglycemia**

WTP: US$77,679

**
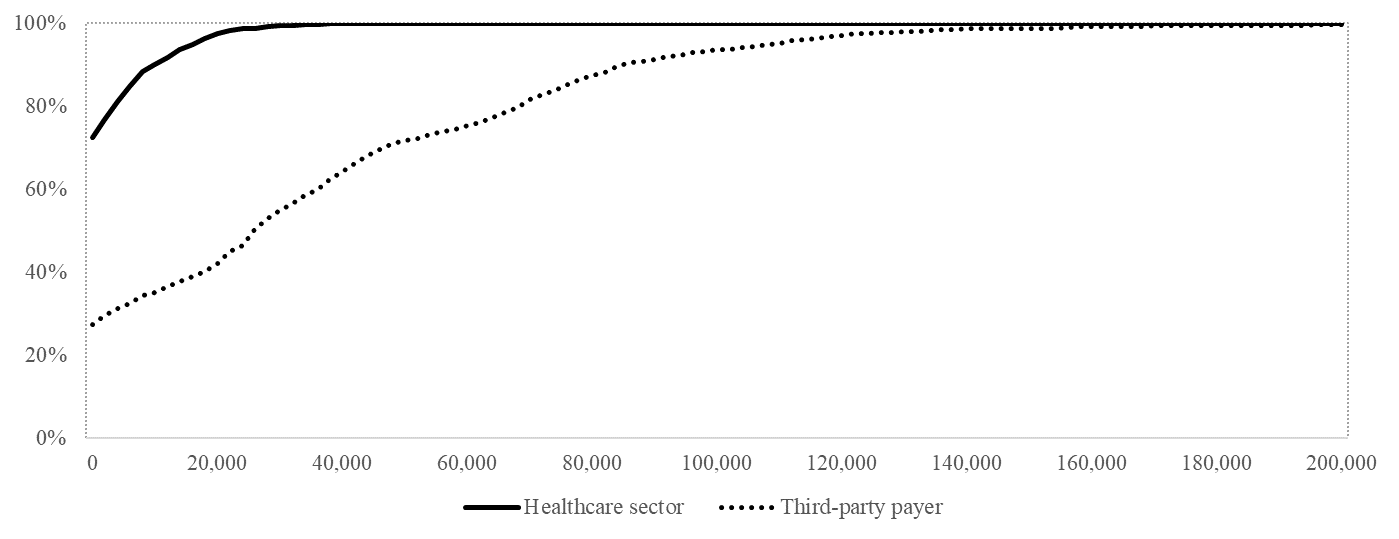
**

Abbreviation: WTP, willingness to pay.

Note: All costs are presented in 2019 US dollars.

**Table S5: Description of existing studies on cost-effectiveness of glucagon-like peptide-1 receptor agonists (GLP-1RAs) versus insulin therapy**

| **Study**  **(country, perspective)** | **Data source (effect, cost, utility), model** | **Study population (age, baseline treatment, T2D cohort type)** | **Time**  **horizon, discount rate (for both effectiveness and cost)** | **Intervention** | **Comparator** | **Results (cost in 2019 US dollars)** | **Main conclusion** |
| --- | --- | --- | --- | --- | --- | --- | --- |
| **Exenatide** | | | | | | | |
| Edwards, 2006^1^  (US, not available) | **Efficacy/effectiveness:** a phase III trial of exenatide (26-week follow-up)^2^ and a phase III trial of NPH insulin (24-week follow-up)^3^  **Cost and utility:**  Cost: United States average wholesale prices (only considered drug cost)  **Utility**: not available  **Model:** not available | **Age**: 59.8 years  **Baseline treatment**: metformin+ sulfonylurea (SU)  **Cohort type**: trial | not available, not available | Exenatide | NPH, insulin glargine | **Reduction in HbA1c:**  **Exenatide vs. NPH**  **ICER:** $3,035  **Exenatide vs. glargine.**  **ICER**: -$101,040  **Reductions in body weight:**  **Exenatide vs. NPH**  **ICER:** $362  **Exenatide vs. glargine.**  **ICER**: $197 | Compared to intermediate and long-acting insulin therapies, exenatide does not appear to be as cost effective for the treatment of type 2 diabetes. |
| Ray, 2007^4^  (UK, third-party payer) | **Efficacy/effectiveness:** a phase III trial of exenatide (26-week follow-up)^2^  **Cost and utility:** literature review on the cost and utility modules built in the model with supplementation  **Model:** IMS Core Diabetes Model | **Age:** 58.9 years  **Baseline treatment:** metformin+ SU  **Cohort type:** simulated patients | 35 years, 3.5% | Exenatide | Insulin glargine | **Direct medical costs (**$**):** Exenatide $24,907, insulin glargine $16,510 per patient  **QALYs:** Exenatide 7.39, insulin glargine 6.95  **ICER**: $18,993 per QALY gained | Exenatide is likely to represent good value for money by generally accepted standards in the UK setting in individuals with type 2 diabetes (T2D) inadequately controlled on oral therapy. |
| Woehl, 2008^5^  (UK, third-party payer) | **Efficacy/effectiveness:** a phase III trial of exenatide (26-week follow-up)^2^  **Cost and utility:** literature review on the cost and utility modules built in the model  **Model:** discrete event  simulation (DES) model | **Age:** 59 years  **Baseline treatment:** metformin + SU  **Cohort type:** simulated patients | 40 years, 3.5% | Exenatide | Insulin glargine | **Direct medical costs (**$**):** Exenatide $10,331, insulin glargine $6,581 per patient  **QALYs:** Exenatide 7.68, insulin glargine 7.86  **ICER**: -$20,672 per QALY gained | With current clinical evidence, exenatide **does not** appear to represent a **cost-effective** treatment option for patients with T2D when compared to insulin glargine.  Analysis of cost-effectiveness from a third-party perspective suggests that exenatide is likely to represent good value for money in the German setting. |
| Mittendorf, 2009^6^  (German, third-party payer) | **Efficacy/effectiveness:** a phase III trial of exenatide (26-week follow-up)^2^  **Cost and utility:** literature review on the cost and utility modules built in the model with supplementation  **Model:** IMS Core Diabetes Model | **Age:** 58.9 years  **Baseline treatment:** metformin+SU  **Cohort type:** simulated patients | 10 years, 5% | Exenatide | Insulin glargine | **Direct medical costs (**$**):** Exenatide $22,879, insulin glargine $18,889 per patient  **QALYs** Exenatide 4.87, insulin glargine 4.59  **ICER**: $14,234 per QALY gained |  |
| Beaudet, 2011^7^  (UK, third-party payer) | **Efficacy/effectiveness:** a phase III trial of exenatide (26-week follow-up)^8^ (DURATION-3)  **Cost and utility:** literature review on the cost and utility modules built in the model with supplementation  **Model:** IMS Core Diabetes Model | **Age:** 58 years  **Baseline treatment:** metformin or  metformin + SU  **Cohort type:** simulated patients | 50 years, 3.5% | Exenatide | Insulin glargine | **Direct medical costs (**$**):** Exenatide $18,260, insulin glargine $16,620 per patient  **QALYs** Exenatide 8.03, insulin glargine 7.85  **ICER**: $8,979 per QALY gained | At the prices investigated, the cost per QALY gained for exenatide when compared with insulin glargine in type 2 diabetes patients in the UK setting, was within the range normally. considered cost-effective by NICE. |
| Gaebler, 2012^9^  (US, third-party payer) | **Efficacy/effectiveness:** a phase III trial of exenatide (104, 52, 26, and 26-week follow-up)^8,10-12^  (DURATION-1,2,3, and 5)  **Cost and utility:** Medicare data, Drugstore.com, and literature review on the cost and utility modules built in the model with supplementation  **Model:** Archimedes model | **Age:** 57 years  **Baseline treatment:** metformin or  metformin + SU  **Cohort type:** simulated patients from the National Health and Nutrition Examination Survey (NHANES) 1999–2006 in United States. | 20 years, undiscounted | Exenatide | Moderate-adherence insulin | **Exenatide vs. moderate-adherence insulin**  **Direct medical costs ($):** Exenatide $191,362, moderate-adherence insulin $197,713 per patient  **QALYs**: Exenatide 13.72, moderate -adherence insulin 13.44  **ICER**: -$22,682 per QALY gained | This long-term simulation demonstrated that exenatide treatment may decrease rates of cardiovascular and some microvascular complications of T2D. Increased QALYs and decreased costs were also projected. |
| Samyshkin, 2012^13^  (US, third-party payer) | **Efficacy/effectiveness:** phase-III trial of exenatide (3-year follow-up)^14^  (DURATION-3)  **Cost and utility:** literature review on the cost and utility modules built in the model with supplementation  **Model:** IMS Core Diabetes Model | **Age:** 57.9 years  **Baseline treatment:** metformin or  metformin + SU  **Cohort type:** simulated patients | 35 years, 3% | Exenatide | Insulin glargine | **Direct medical costs ($):** Exenatide $99,291, insulin glargine $94,269 per patient  **QALYs**: Exenatide 8.476, insulin glargine 8.231  **ICER**: $20,448 per QALY gained | Treatment with exenatide (once weekly) is projected to be cost-effective compared to treatment with insulin glargine. |
| Fonseca, 2013^15^  (Spain, third-party payer) | **Efficacy/effectiveness:** a phase III trial of exenatide (26-week follow-up)^8^  (DURATION-3)  **Cost and utility:** literature review on the cost and utility modules built in the model with supplementation  **Model:** IMS Core Diabetes Model | **Age:** 57.9 years  BMI ≥30 kg/m^2^  **Baseline treatment:** metformin or  metformin + SU  **Cohort type:** simulated patients | 35 years, 3% | Exenatide | Insulin glargine | **Direct medical costs ($):** Exenatide $42,522, insulin glargine $40,510 per patient  **QALYs**: Exenatide 8.712, insulin glargine 8.534  **ICER**: $11,288 per QALY gained | The analyses indicate that exenatide is a cost-effective option for the treatment of T2D patients in Spain for patients with a BMI > 30 kg/m^2^ considering a willingness-to-pay threshold of €30,000 per QALY gained |
| Deng, 2015^16^  (China, societal) | **Efficacy/effectiveness:** meta-analysis of three trials  **Cost and utility:** literature review on the cost and utility modules built in the model with supplementation  **Model:** Cardiff Diabetes Model | **Age:** 51.74 years  **Baseline treatment:** oral antidiabetic drugs (OADs)  **Cohort type:** simulated patients | 40 years, 3% | Exenatide | Insulin glargine | **Direct medical costs ($):** Exenatide $49,422, insulin glargine $70,716 per patient  **QALYs**: Exenatide 13.48, insulin glargine 11.60  **ICER**: -$11,466 per QALY gained | Compared with insulin glargine QD, exenatide BID as add-on therapy to OADs is a cost-effective treatment in Chinese patients inadequately controlled by OADs treatments. |
| Gordon, 2016^17^  (Swedish, healthcare sector) | **Efficacy/effectiveness:** a phase III trial of exenatide^18^  **Cost and utility:** literature review on the cost and utility modules built in the model with supplementation  **Model:** Cardiff Diabetes Model | **Age:** 59.80 years  **Baseline treatment:** Insulin glargine  **Cohort type:** simulated patients | 40 years, 3% | Exenatide | Insulin lispro | **Direct medical costs (**$**):**  Exenatide $44,768, insulin lispro $43,256 per patient  **QALYs:** Exenatide 11.51, insulin lispro 10.86  **ICER**: $1,982 per QALY gained | From the Swedish healthcare perspective, analysis with the Cardiff Diabetes Model demonstrated that exenatide BID represents a cost-effective treatment alternative to lispro TID as add-on therapy in T2D patients insufficiently controlled on basal insulin. |
| Gu, 2017^19^  (China, third-party payer) | **Efficacy/effectiveness:** meta-analyses  **Cost:** Literature review and claims data of a T2D cohort with 639 patients aged ≥18 years 2014-2015 in China  **utility:** literature review on the cost and utility modules built in the model with supplementation  **Model:** Cardiff Diabetes Model | **Age:** 50.59 years  **Baseline treatment:** OADs  **Cohort:** simulated patients | 40 years, 3% | Exenatide | Insulin glargine | **Direct medical costs ($):** Exenatide $44,515, insulin glargine $66,387 per patient  **QALYs:** Exenatide 14.26, insulin glargine 12.33  **ICER**: -$11,291 per QALY gained | In Chinese patients with T2D inadequately controlled by OADs, exenatide twice daily is a cost-effective add-on therapy alternative to insulin glargine once daily. |
| Tzanetakos, 2018^20^  (Greece, third-party payer) | **Efficacy/effectiveness:** phase-III trial of exenatide (3-year follow-up)^14^ (DURATION-3)  **Costs and utility:** literature review on the cost and utility modules built in the model with supplementation  **Model:** Cardiff Diabetes Model | **Age:** 57.90 years  **Baseline treatment:** metformin or  metformin + SU  **Cohort type:** simulated patients | 40 years, 3.5% | Exenatide | Insulin glargine | **Direct medical costs ($):**  Exenatide $24,716, insulin glargine $22,715 per patient  **QALYs:** Exenatide 11.94, insulin glargine 11.48  **ICER**: $4,368 per QALY gained | Exenatide (once weekly) was estimated to be cost effective relative to insulin glargine or Lira 1.2 mg for the treatment of T2D in adults not adequately controlled on OAD therapy in Greece. |
| **Lixisenatide** | | | | | | | |
| Huetson, 2015^21^  (Norway, societal) | **Efficacy/effectiveness:** a phase III trial of Lixisenatide (24-week follow-up)^22^  (GetGoal-L trial)  **Cost and utility:** literature review on the cost and utility modules built in the model with supplementation  **Model:** IMS Core Diabetes Model | **Age:** 57.2 years  **Baseline treatment:** basal insulin  **Cohort type:** simulated patients | 45 years, 4% | Lixisenatide | Bolus insulin | **Total lifetime costs ($):** Lixisenatide $258,364, bolus insulin $259,782 per patient  **QALYs:** Lixisenatide 6.908, bolus insulin 6.842  **ICER**: -$21,488 per QALY gained | Lixisenatide may be considered an economically efficient therapy in combination with basal insulin in the Norwegian setting, owing to **cost savings**, weight loss, and associated gains in health-related quality of life. |
| Men, 2020^23^  (China, third-party payer) | **Efficacy/effectiveness:** meta-analyses  **Cost:** literature review on the cost and utility modules built in the model with supplementation  **Model:** IQVIA CORE Diabetes Model | **Age:** 54.8 years  **Baseline treatment:** metformin or  metformin + SU  **Cohort type:** simulated patients | Lifetime, 3% | Lixisenatide | Basal insulin, premixed insulin, placebo | **Lixisenatide vs. basal insulin:**  **Direct medical costs ($):**  Exenatide $60,784, basal insulin $59,916 per patient  **QALYs:** Exenatide 11.13, basal insulin 11.04  **ICER**: $9,478 per QALY gained  **Lixisenatide vs. premixed insulin:**  **Direct medical costs ($):**  Exenatide $60,784, premixed insulin $59,345 per patient  **QALYs:** Exenatide 11.13, premixed insulin 11.06  **ICER**: $19,735 per QALY gained  **Lixisenatide vs. placebo:**  **Direct medical costs ($):**  Exenatide $60,784, premixed insulin $60,203 per patient  **QALYs:** Exenatide 11.13, premixed insulin 11.00  **ICER**: $4,342 per QALY gained | For T2D patients inadequately controlled on OADs, lixisenatide was shown to be comparable to basal insulin and premixed insulin in terms of HbA1c and better than both of the latter in terms of both body weight loss and hypoglycemia. Lixisenatide was also a cost-effective treatment option from the perspective of Chinese health insurance. |
| **Dulaglutide** | | | | | | | |
| Lasalvia, 2017^24^ (Colombia, third-party payer) | **Efficacy/effectiveness:** a phase III trial of dulaglutide (52-week follow-up, 26-week follow-up)^25,26^ (AWARD-4, AWARD-6) | **Age:** 55 years  **Baseline treatment:** No restriction on previous diabetes treatment  **Cohort type:** simulated patients | 5 years, 5% | Dulaglutide | Insulin glargine | **Direct medical costs (**$**):** Dulaglutide $9,631, insulin glargine $6,452 per patient  **QALYs:** Dulaglutide 3.311, insulin glargine 3.156  **ICER**: $20,511 per QALY gained | Its ICER is, however, greater than the accepted threshold for Colombia in base case compared with glargine. |
| Ishii, 2018^27^  (Japan, healthcare sector) | **Efficacy/effectiveness:** a phase III trial of dulaglutide (26-week follow-up)  **Cost and utility:** literature review on the cost and utility modules built in the model with supplementation  **Model:** IQVIA CORE Diabetes Model | **Age:** 56.83 years  **Baseline treatment:** metformin or  metformin+SU  **Cohort type:** simulated patients | 50 years, 2% | Dulaglutide | Insulin glargine | **Direct medical costs (**$**):** Dulaglutide $92,406, insulin glargine $91,222 per patient  **QALYs:** Dulaglutide 15.304, insulin glargine 14.995  **ICER**: $3,837 per QALY gained | Dulaglutide 0.75 mg may be a cost-effective treatment alternative to insulin glargine for patients with T2D in Japan. |
| Pollock, 2019^28^  (Canada, healthcare sector) | **Efficacy/effectiveness:** a phase III trial of dulaglutide (AWARD-2 trial)  **Cost and utility:** literature review on the cost and utility modules built in the model with supplementation  **Model:** Microsoft Excel model (closely following the model structure of the UKPDS OM) | **Age:** 56.66 years  **Baseline treatment:** metformin+SU  **Cohort type:** simulated patients | 40 years, 1.5% | Dulaglutide | Insulin glargine | **Direct medical costs (**$**):** Dulaglutide $75,205, insulin glargine $59,155 per patient  **QALYs:** Dulaglutide 12.90, insulin glargine 12.52  **ICER**: $42,679 per QALY gained | Dulaglutide 1.5 mg would likely be cost-effective relative to insulin glargine in patients with T2D inadequately controlled on metformin and SU in Canada. |
| **Albiglutide** | | | | | | | |
| Bruhn, 2016^29^  (US, third-party payer) | **Efficacy/effectiveness:** a phase III trial of albiglutide^30,31^  (HARMONY-6 and 4 trial)  **Cost and utility:** literature review on the cost and utility modules built in the model with supplementation  **Model:** The Centre for Outcomes Research (CORE) Diabetes Mode | **Age:**   - 55.6 years (albiglutide vs. insulin lispro) - 55.7 years (albiglutide vs. insulin glargine)   **Baseline treatment:**   - basal insulin ± OADs (albiglutide vs. insulin lispro) - metformin ± SU (albiglutide vs. insulin glargine)   **Cohort type:** simulated patients | 50 years, 3% | Albiglutide | Insulin lispro, insulin glargine | **Albiglutide vs. insulin lispro:**  **Direct medical costs (**$**):**  Albiglutide $173,973, insulin lispro $169,012 per patient  **QALYs:** Albiglutide 8.707, insulin lispro 8.608  **ICER**: $49,855 per QALY gained  **Albiglutide vs. insulin glargine:**  **Direct medical costs (**$**):**  Albiglutide $156,754, insulin glargine $153,780 per patient  **QALYs:** Albiglutide 9.141, insulin glargine 9.108  **ICER**: $90,646 per QALY gained | Albiglutide represents a reasonable treatment option for patients with T2D based on its cost utility relative to those of insulin lispro and insulin glargine. |
| **Semaglutide** | | | | | | | |
| Hunt, 2019^32^  (Netherlands, societal) | **Efficacy/effectiveness:** a phase III trial of senaglutide^33^ (30-week follow-up) (SUSTAIN-4 trial)  **Cost and utility:** literature review on the cost and utility modules built in the model with supplementation  **Model:** IQVIA CORE Diabetes Model | **Age:** 56 years  **Baseline treatment:** metformin or  metformin+SU  **Cohort type:** simulated patients | Lifetime, 1.5% (benefit) and 4% (cost) | Semaglutide | Insulin glargine | **Semaglutide 0.5 mg vs. insulin glargine:**  **Direct medical costs (**$**):**  Semaglutide $44,379, insulin glargine $43,480 per patient  **QALYs:** Semaglutide 12.05, insulin glargine 11.85  **ICER**: $4,724 per QALY gained  **Semaglutide 1 mg vs. insulin glargine:**  **Direct medical costs (**$**):**  Semaglutide $43,606, insulin glargine $43,480 per patient  **QALYs:** Semaglutide 12.12, insulin glargine 11.85  **ICER**: $469 per QALY gained | Once-weekly semaglutide is cost-effective versus insulin glargine U100 for the treatment of T2D and represents a good use of healthcare resources in the Netherlands. |
| **Not Specified** | | | | | | | |
| Kiadaliri, 2014^34^  (Sweden, societal) | **Efficacy/effectiveness:** Swedish national diabetes register  **Cost and utility:** literature review on the cost and utility modules built in the model with supplementation  **Model:** Swedish Institute for Health Economics Model: Cohort Model for T2D (IHECM-T2DM) | **Age:** 64.7 years  **Baseline treatment:** metformin  **Cohort type:** simulated patients | 35 years, 3% | GLP-1RA  (liraglutide, exenatide) | NPH insulin | **Direct medical costs (**$**)** Exenatide $383,650, insulin glargine $376,413 per patient  **QALYs:** GLP-1RA 4.75, NPH Insulin 4.50  **ICER**: $28,490 per QALY gained | Assuming a WTP of SEK 500,000 per QALY, treatment strategy with GLP-1RA is a cost-effective strategy in comparison to DPP-4 inhibitors or NPH insulin among T2D patients inadequately controlled with metformin alone in a Swedish setting. |
| The present study, 2020  (Taiwan, third-party payer and healthcare sector) | **Efficacy/effectiveness:** Taiwan’s National Health Insurance Research Database (NHIRD)  **Cost and utility:** individual-level cost data were calculated using NHIRD’s claims and utility data were not available  **Model:** real-world study-based cost-effectiveness analysis | **Age:** 49.4 years  **Baseline treatment:** Multiple glucose-lowering agents  **Cohort type:** T2D patients in Taiwan | mean follow-up: 2.3 years, not applicable | GLP-1RA  (liraglutide, exenatide) | NPH insulin, insulin glargine, insulin determir, premixed insulin human/analogue, short-acting insulin, rapid-acting insulin | **Reduction in all-cause death:** NNT=57  **GLP-1RA vs. insulin from third-party payer perspective**  **ICER:** $54,851 per case of all-cause death prevented  **GLP-1RA vs. insulin from healthcare sector perspective**  **ICER**: -$19,391 per case of all-cause death prevented  **Reduction in hospitalized hypoglycemia:** NNT=30  **GLP-1RA vs. insulin from third-party payer perspective**  **ICER:** $29,115 per case of hospitalized hypoglycemia prevented  **GLP-1RA vs. insulin from healthcare sector perspective**  **ICER**: -$10,293 per case of hospitalized hypoglycemia prevented | Compared to insulin, GLP-1RA was cost-effective or even cost-saving for T2D patients in Taiwan. |

Abbreviations: T2D, type 2 diabetes; US, United States; UK, United Kingdom; IMS, Intercontinental Marketing Statistics; QALY, quality-adjusted life year; ICER, incremental cost-effectiveness ratio; DPP-4 inhibitors, dipeptidyl peptidase-4 inhibitor; NPH, Neutral Protamine Hagedorn; BMI, body mass index; NNT, number needed to treat.

**Summary of studies that were included in the systematic review:**

Nine studies for the cost-effectiveness of exenatide versus insulin glargine utilized treatment efficacy data derived from the drug’s phase III trials [2, 14], and the country-specific cost data or/and utility data or the literature-data-based cost or utility modules built in the models were applied in these studies. Eight of these studies from the United Kingdom [4, 7], Germany [6], United States [13], Greece [20], Spain [15], China [16], and Swedish [17] yielded a consistent conclusion, namely that compared to insulin glargine, the use of exenatide yielded improved QALYs and higher direct medical costs, and the resulting ICERs were lower than the pre-specific willingness-to-pay thresholds, suggesting that exenatide is cost-effective relative to insulin glargine. Cost-saving results of using exenatide versus insulin were also shown in two studies from the United States [9] and China [19], respectively. However, another study from the United Kingdom by Woehl et al [5]. showed that exenatide versus insulin glargine was not cost-effective due to a higher incremental medical cost and a lower QALY gained in a lifetime simulation.

A Norwegian study based on trial efficacy data and country-specific cost and utility data reported the cost-saving result of lixisenatide versus bolus insulin added-on to basal insulin among T2D patients [21], and a study from China showed that lixisenatide was cost-effective compared to insulin (basal and premixed) or placebo [23]. Three studies on the cost-effectiveness of dulaglutide versus insulin glargine from Colombia [24], Japan [27], and Canada [28] based on phase III trial efficacy data and country-specific cost and utility data showed that dulaglutide relative to insulin glargine was cost-effective in inadequately controlled T2D patients treated with metformin and sulfonylurea.

Two studies from the United States [29] and Netherlands [32] for cost-effectiveness of albiglutide and semaglutide, respectively, suggested that these two drugs were cost-effective compared to insulin therapy (insulin lispro or insulin glargine). Kiadaliri et al [34]. assessed the cost-effectiveness of GLP-1RAs treatment (including liraglutide and exenatide) compared to NPH insulin based on the treatment effectiveness data from the Swedish National Diabetes Register and country-specific cost and utility data for T2D patients in a Swedish healthcare setting. They reported that a GLP-1RA treatment strategy was cost-effective in comparison to NPH insulin.

**References:**

1. Edwards KL, Irons BK, Xu T. Cost-effectiveness of intermediate or long-acting insulin versus Exenatide in type 2 diabetes mellitus patients not optimally controlled on dual oral diabetes medications. *Pharm Pract (Granada).* 2006;4(3):129-133.

2. Heine RJ, Van Gaal LF, Johns D, Mihm MJ, Widel MH, Brodows RG. Exenatide versus insulin glargine in patients with suboptimally controlled type 2 diabetes: a randomized trial. *Ann Intern Med.* 2005;143(8):559-569.

3. Riddle MC, Rosenstock J, Gerich J. The treat-to-target trial: randomized addition of glargine or human NPH insulin to oral therapy of type 2 diabetic patients. *Diabetes Care.* 2003;26(11):3080-3086.

4. Ray JA, Boye KS, Yurgin N, et al. Exenatide versus insulin glargine in patients with type 2 diabetes in the UK: a model of long-term clinical and cost outcomes. *Curr Med Res Opin.* 2007;23(3):609-622.

5. Woehl A, Evans M, Tetlow AP, McEwan P. Evaluation of the cost effectiveness of exenatide versus insulin glargine in patients with sub-optimally controlled type 2 diabetes in the United Kingdom. *Cardiovasc Diabetol.* 2008;7:24.

6. Mittendorf T, Smith-Palmer J, Timlin L, Happich M, Goodall G. Evaluation of exenatide vs. insulin glargine in type 2 diabetes: cost-effectiveness analysis in the German setting. *Diabetes Obes Metab.* 2009;11(11):1068-1079.

7. Beaudet A, Palmer JL, Timlin L, et al. Cost-utility of exenatide once weekly compared with insulin glargine in patients with type 2 diabetes in the UK. *J Med Econ.* 2011;14(3):357-366.

8. Diamant M, Van Gaal L, Stranks S, et al. Once weekly exenatide compared with insulin glargine titrated to target in patients with type 2 diabetes (DURATION-3): an open-label randomised trial. *Lancet.* 2010;375(9733):2234-2243.

9. Gaebler JA, Soto-Campos G, Alperin P, et al. Health and economic outcomes for exenatide once weekly, insulin, and pioglitazone therapies in the treatment of type 2 diabetes: a simulation analysis. *Vasc Health Risk Manag.* 2012;8:255-264.

10. Taylor K, Gurney K, Han J, Pencek R, Walsh B, Trautmann M. Exenatide once weekly treatment maintained improvements in glycemic control and weight loss over 2 years. *BMC Endocr Disord.* 2011;11:9.

11. Wysham C, Bergenstal R, Malloy J, et al. DURATION-2: efficacy and safety of switching from maximum daily sitagliptin or pioglitazone to once-weekly exenatide. *Diabet Med.* 2011;28(6):705-714.

12. Blevins T, Pullman J, Malloy J, et al. DURATION-5: exenatide once weekly resulted in greater improvements in glycemic control compared with exenatide twice daily in patients with type 2 diabetes. *J Clin Endocrinol Metab.* 2011;96(5):1301-1310.

13. Samyshkin Y, Guillermin AL, Best JH, Brunell SC, Lloyd A. Long-term cost-utility analysis of exenatide once weekly versus insulin glargine for the treatment of type 2 diabetes patients in the US. *J Med Econ.* 2012;15 Suppl 2:6-13.

14. Diamant M, Van Gaal L, Guerci B, et al. Exenatide once weekly versus insulin glargine for type 2 diabetes (DURATION-3): 3-year results of an open-label randomised trial. *Lancet Diabetes Endocrinol.* 2014;2(6):464-473.

15. Fonseca T, Clegg J, Caputo G, Norrbacka K, Dilla T, Alvarez M. The cost-effectiveness of exenatide once weekly compared with exenatide twice daily and insulin glargine for the treatment of patients with type two diabetes and body mass index ≥30 kg/m(2) in Spain. *J Med Econ.* 2013;16(7):926-938.

16. Deng J, Gu S, Shao H, Dong H, Zou D, Shi L. Cost-effectiveness analysis of exenatide twice daily (BID) vs insulin glargine once daily (QD) as add-on therapy in Chinese patients with Type 2 diabetes mellitus inadequately controlled by oral therapies. *J Med Econ.* 2015;18(11):974-989.

17. Gordon J, McEwan P, Sabale U, Kartman B, Wolffenbuttel BH. The cost-effectiveness of exenatide twice daily (BID) vs insulin lispro three times daily (TID) as add-on therapy to titrated insulin glargine in patients with type 2 diabetes. *J Med Econ.* 2016;19(12):1167-1174.

18. Diamant M, Nauck MA, Shaginian R, et al. Glucagon-Like Peptide 1 Receptor Agonist or Bolus Insulin With Optimized Basal Insulin in Type 2 Diabetes. *Diabetes Care.* 2014;37(10):2763-2773.

19. Gu S, Wang X, Qiao Q, Gao W, Wang J, Dong H. Cost-effectiveness of exenatide twice daily vs insulin glargine as add-on therapy to oral antidiabetic agents in patients with type 2 diabetes in China. *Diabetes Obes Metab.* 2017;19(12):1688-1697.

20. Tzanetakos C, Bargiota A, Kourlaba G, Gourzoulidis G, Maniadakis N. Cost Effectiveness of Exenatide Once Weekly Versus Insulin Glargine and Liraglutide for the Treatment of Type 2 Diabetes Mellitus in Greece. *Clin Drug Investig.* 2018;38(1):67-77.

21. Huetson P, Palmer JL, Levorsen A, Fournier M, Germe M, McLeod E. Cost-effectiveness of once daily GLP-1 receptor agonist lixisenatide compared to bolus insulin both in combination with basal insulin for the treatment of patients with type 2 diabetes in Norway. *J Med Econ.* 2015;18(8):573-585.

22. Riddle MC, Aronson R, Home P, et al. Adding once-daily lixisenatide for type 2 diabetes inadequately controlled by established basal insulin: a 24-week, randomized, placebo-controlled comparison (GetGoal-L). *Diabetes Care.* 2013;36(9):2489-2496.

23. Men P, Qu S, Song Z, Liu Y, Li C, Zhai S. Lixisenatide for Type 2 Diabetes Mellitus Patients Inadequately Controlled on Oral Antidiabetic Drugs: A Mixed-Treatment Comparison Meta-analysis and Cost–Utility Analysis. *Diabetes Therapy.* 2020;11(8):1745-1755.

24. Lasalvia P, Baquero L, Otálora-Esteban M, Castañeda-Cardona C, Rosselli D. Cost Effectiveness of Dulaglutide Compared with Liraglutide and Glargine in Type 2 Diabetes Mellitus Patients in Colombia. *Value Health Reg Issues.* 2017;14:35-40.

25. Blonde L, Jendle J, Gross J, et al. Once-weekly dulaglutide versus bedtime insulin glargine, both in combination with prandial insulin lispro, in patients with type 2 diabetes (AWARD-4): a randomised, open-label, phase 3, non-inferiority study. *Lancet.* 2015;385(9982):2057-2066.

26. Dungan KM, Povedano ST, Forst T, et al. Once-weekly dulaglutide versus once-daily liraglutide in metformin-treated patients with type 2 diabetes (AWARD-6): a randomised, open-label, phase 3, non-inferiority trial. *Lancet.* 2014;384(9951):1349-1357.

27. Ishii H, Madin-Warburton M, Strizek A, Thornton-Jones L, Suzuki S. The cost-effectiveness of dulaglutide versus insulin glargine for the treatment of type 2 diabetes mellitus in Japan. *J Med Econ.* 2018;21(5):488-496.

28. Pollock RF, Norrbacka K, Cameron C, Mancillas-Adame L, Jeddi M. A cost-utility analysis of dulaglutide versus insulin glargine as third-line therapy for Type 2 diabetes in Canada. *J Comp Eff Res.* 2019;8(4):229-240.

29. Bruhn D, Martin AA, Tavares R, Hunt B, Pollock RF. Cost-utility of albiglutide versus insulin lispro, insulin glargine, and sitagliptin for the treatment of type 2 diabetes in the US. *J Med Econ.* 2016;19(7):672-683.

30. Rosenstock J, Fonseca VA, Gross JL, et al. Advancing basal insulin replacement in type 2 diabetes inadequately controlled with insulin glargine plus oral agents: a comparison of adding albiglutide, a weekly GLP-1 receptor agonist, versus thrice-daily prandial insulin lispro. *Diabetes Care.* 2014;37(8):2317-2325.

31. Weissman PN, Carr MC, Ye J, et al. HARMONY 4: randomised clinical trial comparing once-weekly albiglutide and insulin glargine in patients with type 2 diabetes inadequately controlled with metformin with or without sulfonylurea. *Diabetologia.* 2014;57(12):2475-2484.

32. Hunt B, Malkin SJP, Moes RGJ, Huisman EL, Vandebrouck T, Wolffenbuttel BHR. Once-weekly semaglutide for patients with type 2 diabetes: a cost-effectiveness analysis in the Netherlands. *BMJ Open Diabetes Res Care.* 2019;7(1):e000705.

33. Aroda VR, Bain SC, Cariou B, et al. Efficacy and safety of once-weekly semaglutide versus once-daily insulin glargine as add-on to metformin (with or without sulfonylureas) in insulin-naive patients with type 2 diabetes (SUSTAIN 4): a randomised, open-label, parallel-group, multicentre, multinational, phase 3a trial. *Lancet Diabetes Endocrinol.* 2017;5(5):355-366.

34. Kiadaliri AA, Gerdtham UG, Eliasson B, Carlsson KS. Cost-utility analysis of glucagon-like Peptide-1 agonists compared with dipeptidyl peptidase-4 inhibitors or neutral protamine hagedorn Basal insulin as add-on to metformin in type 2 diabetes in sweden. *Diabetes Ther.* 2014;5(2):591-607.
